# Collaboration Between Oregon’s Chronic Disease Programs and Medicaid to Decrease Smoking Among Medicaid-Insured Oregonians With Asthma

**Published:** 2005-10-15

**Authors:** R. David Rebanal, Richard Leman

**Affiliations:** Oregon Health Services, Health Promotion and Chronic Disease Prevention Program; Mr. Rebanal is also affiliated with the Centers for Disease Control and Prevention, Atlanta, Ga, and was affiliated with the Oregon Health Services, Health Promotion and Chronic Disease Prevention Program, Portland, Ore, when this research was conducted.; Oregon Department of Human Services, Office of Disease Prevention and Epidemiology, Health Promotion and Chronic Disease Prevention Program, Portland, Ore.

## Abstract

**Background:**

Environmental tobacco smoke is a leading environmental asthma trigger and has been linked to the development of asthma in children and adults. Smoking cessation and reduced exposure to secondhand tobacco smoke are key components of asthma management. We describe a partnership involving two state agencies and 14 health plans; the goal of the partnership was to decrease smoking and exposure to environmental tobacco smoke among Medicaid-insured Oregonians with asthma.

**Context:**

Oregon's asthma rate is higher than that of the national population, and approximately one third of Oregonians with asthma smoke. The Health Promotion and Chronic Disease Prevention Program (HPCDP) in the Oregon Department of Human Services has collaborated with the Office of Medical Assistance Programs (OMAP) to promote preventive care at the population level.

**Methods:**

Two HPCDP programs — the Oregon Asthma Program and the Oregon Tobacco Prevention and Education Program — worked with OMAP to launch the statewide Asthma–Tobacco Integration Project in 2003. A primary focus of the project is the development of partnerships among health plans, health care providers, and large health care organizations to integrate asthma management and smoking control through systems innovations and provider education. OMAP and its participating health plans also decided to focus cessation efforts on its members with chronic diseases. In addition, HPCDP has collaborated with OMAP to distribute educational tools and information about tobacco's impact on asthma morbidity to Oregon's health care providers who serve low-income Oregonians.

**Consequences:**

The partnership between OMAP and HPCDP program staff members has allowed them to discuss problems, leverage resources, and obtain support for many public health initiatives. In addition, OMAP–HPCDP collaboration on educational workshops and outreach to health care providers has helped convince quality improvement specialists and administrators about the importance of addressing smoking among patients with asthma. The Asthma–Tobacco Integration Project has also led to formative research aimed at increasing community involvement in promoting tobacco-free environments.

**Interpretation:**

Collaboration between HPCDP and OMAP has been an important factor in Oregon's successful smoking cessation efforts in general and in recent efforts to address tobacco use among Oregonians with asthma.

## Background

Asthma is one of the most common chronic diseases in the United States and has a major impact on the quality of life of the individuals who have it, as well as on their families, their friends, and society as a whole. Although no cure for asthma exists, it can be controlled with high-quality medical care and a good self-management plan, including awareness of asthma triggers and how to avoid them. Environmental tobacco smoke is a leading environmental asthma trigger and has been linked to the development of asthma in children and adults (1,2). Among people with asthma, cigarette smoking decreases lung functioning, increases the risk for asthma-related hospital admissions, increases asthma-related health care use, and increases the risk of death from asthma (3,4). Cigarette smoking has also been associated with an impaired therapeutic response to corticosteroids among people with chronic asthma (5). Smoking cessation and reduced exposure to secondhand tobacco smoke are key components of asthma management.

Many state Medicaid programs shifted from fee-for-service systems to predominantly managed care systems in the 1990s, which presented unique opportunities to improve the public's health by integrating disease prevention and public health goals into the health care system (6). In many situations, managed care led to increased monitoring of quality of care and in some systems made reimbursement dependent on performance (7). The Centers for Disease Control and Prevention (CDC) recognized the potential role of managed care in implementing population-level disease prevention activities. The CDC recommended that public health agencies develop partnerships with Medicaid programs to identify cost-effective preventive services for Medicaid populations and hold managed care plans accountable for the delivery of these services (8,9).

We describe a partnership involving two state agencies and 14 health plans. The goal of the partnership was to decrease smoking and exposure to environmental tobacco smoke among Oregonians with asthma who had Medicaid coverage.

## Context

### Asthma and smoking among Medicaid-insured individuals

In Oregon, 9.2% of the adult population has asthma, higher than the national rate of 7.5% (10,11). Approximately 7.3% of Oregon children have asthma, and approximately 2500 asthma-related hospitalizations occur in Oregon each year. Almost 12% of the state's population lives on an income less than the poverty threshold, and 442,000 Oregonians (13%) qualify for Medicaid (12). Among Oregon's population insured by Medicaid, approximately 17% of adults report having asthma (13).

Despite the solid evidence that tobacco smoke is detrimental to the health of people with asthma, data show that 31% of Oregon adults with asthma smoke cigarettes, whereas 23% of Oregon adults without asthma smoke cigarettes (14). Among Oregonians insured by Medicaid, the rate of smoking among adults with asthma is 43% (13). Oregonians who smoke and have asthma report more severe asthma symptoms than Oregonians with asthma who do not smoke. They have more activity limitations, miss more work and school, and seek urgent medical care more often (14).

### Collaboration between Oregon's chronic disease programs and Medicaid

In 1995, the Health Promotion and Chronic Disease Prevention Program (HPCDP) in the Oregon Department of Human Services began working with the Office of Medical Assistance Programs (OMAP), the agency responsible for administering Oregon's Medicaid programs. OMAP is the largest purchaser of managed care in Oregon and has contracts to administer Medicaid with almost all of the major managed care plans in Oregon. Collaboration between HPCDP and OMAP helped the agencies promote preventive care at the population level. Preventive care interventions developed by HPCDP and OMAP were offered to Medicaid-insured patients in managed care settings, which in 1995 comprised 85% of the population on Medicaid and more than a third of Oregon's overall population. In addition, because most health care providers treated at least some Medicaid patients and belonged to one of these major health plans, joint OMAP–HPCDP initiatives had the potential to reach almost all of the primary care physicians in Oregon.

In 1996, HPCDP created a staff position dedicated to exploring potential areas of collaboration between the agency's chronic disease programs and major Oregon health systems. The person in this position works with health system administrators and data personnel to develop standardized measures of asthma care that can be compared across health systems. HPCDP also provided partial funding for an OMAP staff position dedicated to developing and coordinating chronic disease prevention projects of mutual interest.

The coordinated efforts of the people in the two previously described positions contributed to the establishment of a monthly meeting known as the Quality Performance and Improvement Workgroup. Through this workgroup, representatives from all the contracted Medicaid health plans collaborate on prevention activities as part of their OMAP contract. Activities include implementing physician trainings, developing quality performance measures and quality improvement interventions, implementing tracking systems, developing population-based guidelines, and developing health care policy and service reforms. This collaboration helped catalyze a public–private partnership and a public-health–medical partnership that led to coordinated initiatives promoting chronic disease prevention.

One such initiative was the Tobacco Intervention Project, a partnership involving HPCDP, OMAP, and the Tobacco-Free Coalition of Oregon (15). The project was designed to integrate tobacco-use prevention and treatment into routine health care. It resulted in the statewide implementation of a tobacco-cessation program by all Medicaid health plans. The program included counseling and pharmacotherapy, as well as systematic referral to the Oregon Tobacco Quit Line. The program also included an evaluation component so that health plans could conduct patient satisfaction surveys and chart audits, review relevant administrative claims data to assess the program's effects, and obtain data on numbers of Oregon Tobacco Quit Line calls received from their members. Health plan staff members conducted training and outreach programs for their physicians and provided education and outreach programs to members through mass mailings and other forms of communication (15). These initial collaborative projects have led to several more recent joint OMAP–HPCDP initiatives focusing on tobacco and asthma.

## Methods

### Asthma–Tobacco Integration Project

Two HPCDP programs — the Oregon Asthma Program and the Oregon Tobacco Prevention and Education Program — began the statewide Asthma–Tobacco Integration Project in 2003. The goal of the project is to reduce smoking prevalence and secondhand smoke exposure among people with asthma. A primary focus of the project is the development of partnerships among health plans, health care providers, and large health care organizations to integrate asthma management and smoking control through systems innovations and provider education.

One of the strategies for implementing the Asthma–Tobacco Integration Project was to leverage OMAP's investment in tobacco cessation by dedicating HPCDP staff to support OMAP–HPCDP cooperation on asthma management projects, a strategy that had been used previously to promote tobacco cessation. However, because of budget constraints and a hiring freeze, HPCDP was not able to hire new employees. Instead, the state asthma and tobacco programs successfully applied to the CDC's Public Health Prevention Service for a staff member to fill this role. The program sends master's-level, CDC-trained prevention specialists with backgrounds in program management and epidemiology to state and local health departments. This new staff person's duties involve coordination not only between HPCDP's asthma program and tobacco program but also between HPCDP and OMAP.

Discussions between HPCDP and OMAP produced additional tobacco-cessation initiatives at the health systems level. Building on the infrastructure established by the Tobacco Intervention Project, OMAP and its participating health plans decided to target cessation efforts toward its members with chronic diseases. In addition, the health plans in OMAP chose asthma management as a key performance measure for the 2004–2005 fiscal year, partly because of the existence of asthma quality performance indicators developed through a cooperative effort between HPCDP and Oregon's major health plans.

As part of the Asthma–Tobacco Integration Project, HPCDP and OMAP have distributed educational tools and information about tobacco's impact on asthma morbidity to almost all of Oregon's health care providers who serve low-income Oregonians. In April 2004, HPCDP distributed a report on tobacco and asthma in the *CD Summary,* an epidemiology newsletter that is produced semimonthly by the Oregon Department of Human Services and sent to all physicians and nurse practitioners in Oregon. The report, "Tobacco and Asthma — Enough to Take Your Breath Away," discussed the epidemiology of tobacco use among adults with asthma and provided effective clinical interventions and resources for clinicians (16).

As part of another Asthma–Tobacco Integration Project activity, HPCDP and OMAP staff collaborated to plan and conduct a statewide workshop on asthma management issues for medical directors and quality improvement managers from Medicaid health plans. Such workshops are held twice a year by OMAP. Topics are selected by the OMAP Quality Performance and Improvement Workgroup participants with the purpose of enhancing health plan initiatives. The day-long statewide workshop was called "What Does Good Asthma Care Look Like? The Roles of Health Plans and Providers." Topics included best practices and guidelines that promote high-quality asthma care and the roles of providers, health plans, and the public health field in improving population-level asthma care. The workshop included a speech from a national expert on health system strategies to promote effective asthma management, as well as an expert panel comprised of proponents of asthma-related disease registries, disease management, and other successful asthma-related interventions at the health systems level. The importance of systematically assessing and treating tobacco use in clinical settings was emphasized throughout. Clinical asthma and tobacco tools were provided to medical directors and quality improvement coordinators.

### Additional performance improvement interventions

The activities described have led to additional collaborative initiatives to educate and empower health providers and health systems about tobacco control and asthma care. In July 2005, 14 health plans began using an asthma registry (created through a joint effort between HPCDP and the participating health systems) to conduct smoking-cessation activities for members with asthma. The outreach effort involves distribution of materials (previously tested by focus groups) to encourage members who smoke and have asthma to quit, as well as to encourage quitting among plan members who smoke and care for a household member with asthma. The distributed materials contain information about the effects of tobacco smoke on people with asthma, tobacco-cessation assistance, and information about the Oregon Tobacco Quit Line. Health plans are also promoting the Oregon Asthma Resource Bank, a clinically accurate, patient-tested Web site that contains free, easy-to-read, and culturally appropriate asthma education and clinical management tools developed by asthma experts in Oregon.

## Consequences

The partnership between OMAP and HPCDP program staff members has allowed them to discuss problems, leverage resources, and obtain support for many public health initiatives. Despite competing priorities and limited resources, collaboration between the two agencies has allowed each of them to accomplish more in the area of smoking and asthma than either could have alone. Initial partnerships promoting smoking cessation served as a model for the Asthma–Tobacco Integration Project.

Partly as a result of the Asthma–Tobacco Integration Project and other initial programs, 80% of the Oregon Medicaid plans have implemented tobacco-related policy and planning, quality improvement programs, communication initiatives, and clinical delivery systems; 50% of the dental care organizations have done the same.

OMAP–HPCDP collaboration on educational workshops and outreach to health care providers has helped convince quality improvement specialists and administrators about the importance of addressing smoking among patients with asthma. More than 60 people attended the statewide Quality Improvement Workgroup on asthma. All 14 Medicaid health plans were represented, including two Medicaid dental health plans. In previous years, such workshops were attended primarily by quality improvement managers and medical directors, but because of OMAP's collaboration with HPCDP's asthma program, the workshop's participants also included local public health specialists, clinicians, pharmacists, and education specialists. Survey results from the workshop revealed that more than 95% of the respondents either strongly agreed or agreed that the information presented would be useful in their work. Furthermore, 90% of the respondents appreciated the opportunity to network with members of other health plans and public health programs, share best practices, and learn about other interventions to improve the quality of asthma care.

Direct distribution of Asthma–Tobacco Integration Project information to clinicians has had positive results. After HPCDP released its *CD Summary* newsletter on tobacco's impact on people with asthma, several health plans used information from the publication in their own health plan newsletters (16).

The Asthma–Tobacco Integration Project has also led to formative research aimed at increasing community involvement in promoting tobacco-free environments. HPCDP has conducted a series of focus groups among populations with a high smoking prevalence (including various racial and ethnic groups and low socioeconomic status groups) to identify smoking-cessation messages that motivate and are relevant to these populations. Additional populations considered priorities for focus groups include people with asthma who smoke and people who smoke and have a child with asthma. Several health plans helped recruit their members to become focus group participants. The information will be used in targeted media campaigns and other interventions to generate grassroots support for tobacco-cessation and prevention programs.

## Interpretation

Collaboration between HPCDP and OMAP has been an important factor in Oregon's successful smoking cessation efforts in general and in recent efforts to address tobacco use among Oregonians with asthma. Initiatives have been greatly enhanced by the staff positions that were developed to establish and expand collaboration between HPCDP and OMAP and between HPCDP and major health plans. In addition, the collaboration between the two agencies has been driven by a clear common interest: for humanitarian and economic reasons, OMAP has been motivated to decrease smoking prevalence among people with asthma and other chronic diseases — one of the core missions of HPCDP.

The partnership between public health professionals and the administrators and directors of Oregon's Medicaid plans has helped maintain the Medicaid program's focus on chronic disease prevention. In addition, cooperation between public health professionals and OMAP provides an extensive network through which HPCDP can distribute educational tools and messages regarding smoking cessation and chronic disease management to physicians and nurse practitioners throughout Oregon. Additional process and outcome evaluations to assess the effectiveness of recent partnership activities addressing tobacco use and asthma are currently in progress.

The monthly OMAP Quality Performance and Improvement Workgroup has been an effective forum through which HPCDP and OMAP can collaborate with health plans and optimize use of resources and staff time. Public health professionals provide expert information on epidemiology, surveillance, and health promotion program planning. OMAP and the participating health plans serve as leaders and provide the practical knowledge that makes it possible to translate the objectives of an intervention such as the Asthma–Tobacco Integration Project into functional clinical systems. In addition, OMAP encourages health plans and health care practitioners to be accountable for promoting high-quality care.
